# Time to Kidneys Failure Modeling in the Patients at Adama Hospital Medical College: Application of Copula Model

**DOI:** 10.34172/jrhs.2022.84

**Published:** 2022-07-11

**Authors:** Firomsa Shewa, Selamawit Endale, Gurmessa Nugussu, Jaleta Abdisa, Ketema Zerihun, Akalu Banbeta

**Affiliations:** ^1^Department of Statistics, Assosa University, Assosa, Ethiopia; ^2^Department of Statistics, Jimma University, Jimma, Ethiopia; ^3^Department of Statistics, Madda Walabu University, Bale Robe, Ethiopia

**Keywords:** Dependence, Kidney failure, Retrospective study, Time to events

## Abstract

**Background:** Kidney failure is a common public health problem around the world. The vast majority of kidney failure cases in Sub-Saharan African nations, including Ethiopia, go undetected and untreated, resulting in practically certain mortality cases. This study was aimed primarily to model the time to (right and left) kidneys failure in the patients at Adama Hospital Medical College using the copula model.

**Study design:** A retrospective cohort study.

**Methods:** The copula model was used to examine join time to the right and left kidneys failure in the patients by specifying the dependence between the failure times. We employed Weibull, Gompertz, and Log-logistic marginal baseline distributions with Clayton, Gumbel, and Joe Archimedean copula families.

**Results:** This research comprised a total of 431 patients, out of which, 170 (39.4%) of the total patients failed at least one kidney during the follow-up period. Factors such as sex, age, family history of kidney disease, diabetes mellitus, hypertension, and obesity were found to be the most predictive variables for kidney failure in the patients. There was a 41 percent correlation between the patients’ time to the right and left kidneys failure.

**Conclusion:** The patients’ kidney failure risk factors included being a male, older adult, obese, hypertensive, diabetic and also having a family history of kidney disease. The dependence between the patient’s time to the right and left kidneys failure was strong. The best statistical model for describing the kidney failure datasets was the log-logistic-Clayton Archimedean copula model.

## Background

 Kidney failure (end-stage renal disease) is an irreversible condition in which one or both kidneys fail to filter waste materials from the blood adequately.^1^ Glomerular filtration rate (GFR) values that are less than 60 mL/min/1.73 m^2^ for three months or more indicate chronic kidney disease, while GFR values fewer than 15 mL/min/1.73 m^2^, indicate a kidney failure^1^case.

 Kidney failure is a common public health problem around the world, with a growing incidence and prevalence on one hand, and high costs and poor outcomes^2^on the other hand. Kidney failure has increased in frequency and prevalence in the last ten years, and this trend is projected to continue.^3^ In 2016, the United States Renal Data System stated that the kidney failure incidence rates in Taiwan, the United States, the Jalisco region of Mexico, and Thailand were 493, 378, 355, and 346 per million populations per year, respectively.^4^ According to the 2019 Global Renal Health Atlas, 14.5 million people worldwide have kidney failure, and due to the economic, social, and political problems, just 5.4 million of them get treated. Furthermore, each year, over two million people die as a result of a lack of access to healthcare.^5^

 In Africa, kidney illness is at least three to four times more common than in developed countries.^6^ Patients with kidney failure in Africa have the lowest access to the renal replacement therapy, and just nine to 16 percent of them are treated, and the treatment rates in Central and Eastern Africa are estimated to be as low as one to three percent because of the high cost.^7^ Most of the kidney failure cases in Sub-Saharan African nations, including Ethiopia, go undetected and untreated, resulting in practically certain mortality cases.^8^ Ethiopia has a high prevalence of renal disease, which has risen to 12.2 percent in recent years due to an increase in diabetes and hypertension.^9^ According to World Health Organization (WHO) data, published in April 2011, kidney illness claimed the lives of 12 038 Ethiopians, accounting for 1.47 percent of all death cases. According to a study conducted at Adama Hospital Medical College in 2016, 27.40% of 500 patients with end-stage renal disease, died^10^ of this disease.

 In medical studies, it is usual to record two event times for each patient, such as the failure times of the paired human organs.^11^ These types of events are linked because they are from the same subject^12^ and studying such data necessitates some model parameters on the temporal dependence on the bivariate event endpoint.^13^ Traditional survival analysis techniques assume that the survival times of the different subjects are independent. However, as a pair of kidneys share the same biological gene, the patients’ times to the right and left kidneys failure, are not independent of each other. When the event times are dependent on a survival study, completing the analysis using methods based on the independent assumptions leads to inaccurate estimates.

 As a result, we used the copula model which includes the influence of variables on the failure times in the presence of dependence^14^ and deals with two events per subject and dependency between the failure times. This study was aimed primarily to investigate the relationship between a patient’s time to the right and left kidneys failure, as well as the influence of the variables on the dependent structure.

## Methods

###  Study area 

 Adama Hospital Medical College was the site of the research. Hailemariam Mamo Memorial Hospital and Adama Referral Public Hospital were the hospital’s two prior names in the past. It is one of Ethiopia’s first medical hospitals, located in Adama, in Oromia region, 100 km southeast of Addis Ababa. Because of its location, patient load, and staff capacity, the hospital was promoted to a medical college in 2003 E.C.

###  Study design and data collection 

 In this study, a retrospective investigation was carried out. Data were gathered from the patients’ medical records from January 1, 2015, to January 30, 2020, by the health professionals.

###  Study population and variables 

 All kidney disease patients who had registered at Adama Hospital Medical College were included in the study. There were a total of 431 patients considered. The response variables were the patients’ times to the right and left kidneys failure, which were measured over a few days. The starting point was the date on which the kidney disease patients were admitted to the hospital. The study came to an end when the kidney disease patient died or when the study period concluded on January 30, 2020.

 During the study, one of the following four scenarios might occur in a patient: a) [1, 1] both of the patient’s kidneys were failing, b) [1, 0] only the patient’s right kidney was failing, c) [0, 1] only the patient’s left kidney was failing, or d) [0, 0] neither of the patient’s kidneys were failing. In the kidney failure dataset, the times to the right and left kidneys failure in the patients could not be precisely observed, leading to bivariate censored data. Right bivariate censored data occurs when the study ends before the occurrence of one or both events. Death, dropout, referral to another facility, or the termination of the research were all the reasons for censorship. By the way, sex, smoking status, family history, age, alcohol consumption, diabetes mellitus, hypertension, anemia, and obesity were all the explanatory variables.

###  Inclusion and exclusion criteria 

 Patients who had a GFR of less than 60 mL/min/1.73m^2^ and had provided complete information in their registration log books or on their patient identity cards were considered eligible for the trial. Patients who did not provide enough information on one of the critical factors in their registration books or on their identity cards were not eligible. Patients who were born with only one kidney or who were born with two kidneys but only one of them worked were also excluded from the study.

###  Statistical methods

 Thecopula model was used to examine the bivariate event times (join time to the right and left kidneys failure) in the patients by specifying the dependence between the failure times. First, let us show the notation for bivariate time to event data. Assume that there are n patients. Let (*T*_1i_*,T*_2i_) and (*C*_1i_*,C*_2i_), *i* = 1, 2, 3,…, *n* denotes the bivariate failure times and censoring times for the i^th^ patients, respectively. Then for each patient, we observe


$$D_{i}=\left\{(Y_{ji},\Delta_{ji},Z_{ji});Y_{ji}=\min\left(T_{ji},C_{ji}\right),\Delta_{ji}=I\left(T_{ji}\leq C_{ji}\right),j=1,2\right\}$$

where *C*_ji_ is the censoring time, and T_ji_ is the failure time, ∆_ji_ is the censoring indicator and Z_ji_ is the covariate vector. Let $S_{j}\left(t_{j}\right)=P\left(T_{j}>t_{j}\right)$ denotes the marginal survival function and $S\left(t_{1},t_{2}\right)=P\left(T_{1}>t_{1},T_{2}>t_{1}\right)$ denotes the joint survival distribution. Sklar’s theorem^15^ states if the marginal survival functions $S_{1}\left(t_{1}\right)=P\left(T_{1}>t_{1}\right)$ and $S_{2}\left(t_{2}\right)=P\left(T_{2}>t_{2}\right)$ for *T*_1_ and *T*_2_ are continuous, then there exists a unique copula function *C*_η_ in a way that for all *t*_1_*,t*_2_ ≥ 0.


(1) $$S\left(t_{1},t_{2}\right)=C_{\eta }\left\{S_{2}\left(t_{2}\right),S_{1}\left(t_{1}\right)\right\},t_{1},t_{2}\geq 0$$

Copula has provided unified statistical methodologies and flexible survival models. The copula parameter (*η*) is used to show the dependency structure between the times to the right and left kidneys failure in the patients. The copula uniqueness is for the fact that it models two marginal distributions and their dependence separately, allowing the marginal models to be flexible and the covariate effects to be easily interpreted.^16^ The Archimedean copula family, which is one of the most common copula families because of its flexibility and simplicity,^17^ is one of the most popular copula families for bivariate events data. Clayton, Gumbel, and Joe are the most commonly employed Archimedean copula models in the survival analyses.

 Kendall’s tau (τ) is the most commonly utilized measure of the dependence degrees/levels between bivariate event times in practice. Kendall’s tau is just a function of *η* and is dependent only on the copula function, not on the marginal distributions. The Kendall’s tau (τ) for a Clayton copula is given as $\tau =\eta /\left(\eta +2\right)$ . Thus, when >0, meaning times to bivariate events are positively linked, and are independent^18^when *η→0* Similarly, for Gumbel Copula,^19^ $\tau =\frac{\eta }{\eta +1},$ meaning times to bivariate events are positively associated when *η* ≥1 and are independent when =1. For a Joe copula^16^, τ is given by the below equation, and meaning times to bivariate events are positively associated when η ≥ 1 and are independent when η = 1.


(2) $$\tau =\sum_{k=1}^{\infty }\frac{1}{k\left(\eta +2\right)\left\{\eta \left(k-1\right)+2\right\}}$$

It is required to design a regression model for the margins to analyze the influence of the variables on the patients’ time to kidney failure. Proportional hazards models (Weibull and Gompertz), as well as proportional odds (Log-logistic) models are supported by the marginal models.

 Generally, the marginal survival model in terms of the hazard function is given by:


(3) $$\lambda_{j}\left(t_{ji}\left|Z_{ji}\right.\right)=\lambda_{0j}\left(t_{ji}\right)\exp\left(\beta^{\prime }Z_{ji}\right),\;j=1,2,\;i=1,2,...,n$$

Where** λ**_0j_ is the baseline hazard function for the j^th^ margin, Z_ji_ is the covariate for the i^th^ patient, and j^th^ margin and β are the coefficient of the covariates. We used Clayton, Gumbel, and Joe Archimedean copula families with Weibull, Gompertz, and log-logistic marginal baseline distributions.

###  Model selection and diagnostics

 Several model selection procedures have been proposed for the copula-based time to events endpoints models.^21^ Akaike information criterion (AIC) and Bayesian information criterion (BIC) are useful to choose the best fitting copula. AIC and BIC are given by:


(4) $$AIC=-2\log L\left(D;\hat{\theta }\right)+2K$$

(5) $$BIC=-2\log L\left(D;\hat{\theta }\right)+k\ln\left(n\right),$$

Where, k is the number of parameters estimated by the model; n is the number of observations and $L\left(D;\hat{\theta }\right)$ is the joint maximized value of the likelihood function of the model, where $\hat{\theta }$ are the parameter values that maximize the likelihood function. In this study, an innovative two-step estimate technique was used for parameter estimation, which maximum likelihood estimation was the most common method, as well as the Newton method of optimization.

 Regardless of which type of the model is fitted and how the variables are selected to be in the model, it is important to evaluate how well the model fits the data. To check the adequacy of the marginal baseline distribution, the Weibull is plotted by the log of the cumulative hazard with the logarithm of time; the Gompertz is plotted by the log of the hazard with the time, and the log-logistic is plotted by the log-failure odd with the log time.^22^ The plot should resemble a straight line if the baseline marginal distribution assumption holds constant. The scatter plot of the joint survival distribution or scatter plot of the bivariate event times are used to check the adequacy of the Archimedean copula families.^23^ If the scatter plot is condensed, the given Archimedean copula family fits the kidney failure datasets well. R. software (version 4.0.5) with the “CopulaCenR” package was used for the data analysis.^17^

## Results

 This research comprised a total of 431 patients. Out of which, 170 (39.4%) of the patients failed at least one kidney during the follow-up period. Furthermore, 51 (11.8%), 43 (10%), and 76 (17.6%) patients were found to have failed only their right, left, or both kidneys, respectively, whereas 261 (60.6%) were found to have not failed both kidneys during the follow-up period (Table 1). The entire median failure time was 897 days, with 270 and 1080 days being the smallest and greatest observed event times, respectively.

**Table 1:** Descriptive statistics on the number of kidneys failure cases

**Categories **	**Variables**							
	**(0, 0), n=261**		**(1, 0), n=51**		**(0, 1), n=43**		**(1, 1), n=76**	
	**Number**	**Percent**	**Number**	**Percent**	**Number**	**Percent**	**Number**	**Percent**
Sex								
Female	164	38.1	16	3.7	15	3.5	42	9.7
Male	97	22.5	35	8.1	28	6.5	34	7.9
Residence								
Rural	114	26.5	22	5.1	15	3.5	22	5.1
Urban	147	34.1	29	6.7	28	6.5	54	12.5
Smoking								
Nonsmoker	184	42.7	31	7.2	22	5.1	50	11.6
Smoker	77	17.9	20	4.6	21	4.9	26	6.0
Family history								
No	164	38.1	25	5.8	22	5.1	50	11.6
Yes	97	22.5	26	6.0	21	4.9	26	6.0
Age (y)								
≤35	169	39.2	4	0.9	13	3.0	6	1.4
36-55	59	13.7	14	3.2	9	2.1	30	7.0
≥56	33	7.7	33	7.7	21	4.9	40	9.2
Alcohol consumption								
No	162	37.6	25	5.8	15	3.5	45	10.4
Yes	99	23.0	26	6.0	28	6.5	31	7.2
Diabetes mellitus								
No	183	42.4	30	7.0	25	5.8	44	10.2
Yes	78	18.1	21	4.9	18	4.2	32	7.4
Hypertension								
No	142	33.0	17	3.9	13	3.0	21	4.9
Yes	119	27.6	34	7.9	30	7.0	55	12.7
Anemia								
No	173	40.2	30	7.0	24	5.6	40	9.3
Yes	88	20.4	21	4.8	19	4.4	36	8.3
Obesity								
No	179	41.6	9	2.0	20	4.7	28	6.5
Yes	82	19.0	42	9.7	23	5.3	48	11.1

Source: Adama Hospital Medical College, Ethiopia; from 1^st^ January 2015 to 30^th^January 2020. (1, 1): both kidneys; (1, 0): only right kidney; (0, 1): only left kidney; (0, 0): neither left nor right kidneys

 By the way, univariable and multi-variable analyses were applied. In univariable analysis, the model which contains each covariate at a time was ﬁtted to determine variables that have the potential for being included in the multivariable analysis. Covariates in the univariable analysis with *P* values less than 25% were considered for multi-variable analysis.^18^ In the univariable analysis covariates like sex, family history, age (56 years and older), diabetes mellitus, hypertension, anemia, and obesity were signiﬁcant at 25% level of signiﬁcance in all models. However, residence, smoking status, and alcohol consumption were not signiﬁcant at 25% level of signiﬁcance, and they were excluded from the multivariable analysis.

 The Weibull, Gompertz, and log-logistic were used for the parametric marginal baseline distribution; and Clayton, Gumbel, and Joe Archimedean copula families were used in the multivariable survival analysis. The log-logistic-Clayton Archimedean copula model’s AIC and BIC values were 789.20 and 4297.55, respectively, which were the lowest among all models. In addition, the model had a higher Final joint maximum log-likelihood value of -2121.48. As a result, the most efficient model for describing the kidney failure datasets was the log-logistic-Clayton Archimedean copula model. With the Clayton Archimedean copula model, the measure of the parameters’ dependence was at the highest level when we assumed the log-logistic marginal distribution (0.41), followed by the Weibull marginal distribution (0.40) (Table 2).

**Table 2:** The comparisons of the models

**Marginal baseline distribution**	**AIC**	**BIC**	**Final llk**	**τ**
Weibull				
Clayton	4275.54	4312.13	-2128.77	0.40
Gumbel	4282.81	4319.41	-2132.41	0.30
Joe	4289.76	4326.36	-2135.88	0.24
Gompertz				
Clayton	4386.07	4414.53	-2686.03	0.38
Gumbel	4406.38	4434.84	-2696.19	0.18
Joe	4413.64	4442.10	-2699.82	0.13
Log-logistic				
Clayton	4260.95	4297.55	-2121.48	0.41
Gumbel	4277.56	4314.16	-2129.78	0.27
Joe	4285.08	4321.67	-2133.54	0.20

Source: Adama Hospital Medical College, Ethiopia; from 1^st^ January 2015 to 30^th^January 2020 AIC: Akaike information criteria, BIC: Bayesian information criteria, Final llk: Joint maximum log-likelihood, τ: Kendall’s tau

 The findings suggested that sex, family history, age, diabetes, hypertension, and obesity were the most significant predictors of kidney failure in the patients. At a 5% level of significance, the copula parameter (*η*) was significant, implying that the times to the right and left kidneys failure were dependent. The Kendall’s tau (τ), revealed a strong correlation between the times to the right and left kidneys failure in the patients (τ = 41%) (Table 3).

**Table 3:** Multi-variable analysis using the log logistic-Clayton Archimedean copula model (AIC= 4260.95, BIC=4297.55, τ = 0.41)

**Variables**	**Estimate**	**SE**	***P*** ** Value**	**OR (95% CI)**
Sex				
Female	Ref.			
Male	0.37	0.18	0.038	1.45 (1.01, 2.07)
Age (y)				
≤35	Ref.			
36-55	0.04	0.22	0.873	1.04 (0.67, 1.60)
≥56	0.65	0.27	0.015	1.91 (1.13, 3.22)
Family history				
No	Ref.			
Yes	0.42	0.19	0.030	1.53 (1.04, 2.23)
Hypertension				
No	Ref.			
Yes	0.75	0.22	0.001	2.11 (1.36, 3.27)
Diabetes mellitus				
No				
Yes	0.43	0.14	0.003	1.53 (1.16,2.02)
Anemia				
No	Ref.			
Yes	0.18	0.19	0.204	1.20 (0.81, 1.76)
Obesity				
No	Ref.			
Yes	0.41	0.20	0.039	1.50 (1.01, 2.23)
η	1.40	0.27	0.001	

Source: Adama Hospital Medical College, Ethiopia; from 1^st^ January 2015 to 30^th^January 2020 SE: standard error, η: dependence parameter, τ: Kendall’s tau, OR: odd ratio, AIC: Akaike information criteria, BIC: Bayesian information criteria, Final llk: Joint maximum log-likelihood, τ: Kendall’s tau.

 The odds of kidneys failure for the male patients were 45% more than for the female patients (OR=1.45; 95% CI: 1.01, 2.07). The odds of kidney failure for patients with a family history of kidney diseases was 53% more than patients without a family history of kidney diseases (OR=1.53; 95% CI: 1.04, 2.23). The odds of kidney failure for the patients aged 56 years and older were 90% more than those patients aged less than 35 (OR = 1.91; 95% CI: 1.13, 3.22). The odds of the kidney failure for hypertensive patients were twice of the patients who did not have hypertension (OR=2.11; 95% CI: 1.36, 3.27). The odds of kidney failure for a diabetic patient was 53% more than for non-diabetic patients (OR= 1.532; 95% CI: 1.1590, 2.024). The odds of kidney failure for obese patients (BMI above 30 kg/m^2^) were 50% more than for non-obese patients (OR=1.50; 95% CI: 1.01, 2.26).

**Figure 1:**
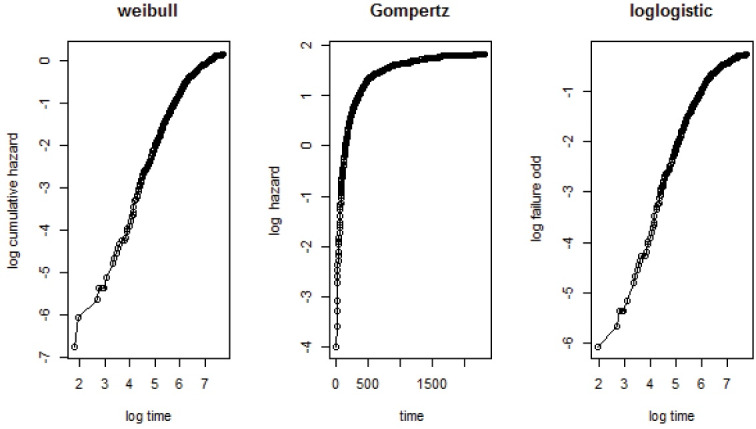
Graphical evaluation of the marginal distributions.

**Figure 2:**
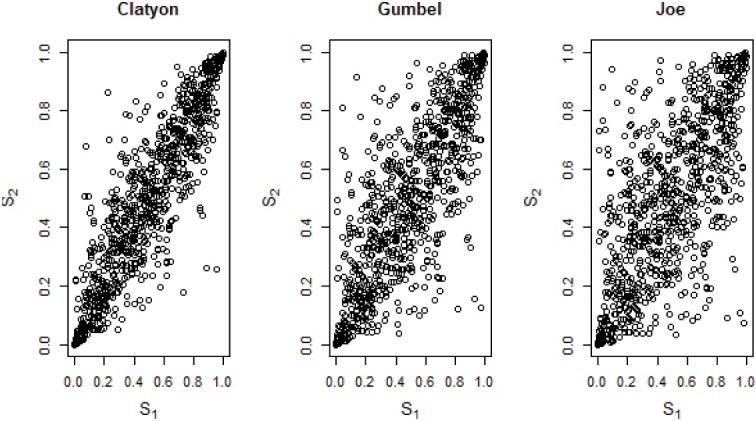
The scatter plots of the joint survival distribution for Archimedean copulas family.

 The graphical evaluation plot was used to check the suitability of the baseline marginal distribution. The specified baseline marginal distribution was appropriate for the datasets if the plot was linear. The log-logistic plot was more linear than the others. The patterns indicated that the log-logistic marginal distribution was fit for the kidney failure datasets (Figure 1). The scatter plot of the joint survival distribution was used to verify the adequacy of the Archimedean copula family. Clayton scatter plots appeared to act more closely or condensed than Gumbel’s and Joe’s. According to the scatter plot (Figure 2), the Clayton copula appeared to fit the kidney failure datasets well.

## Discussion

 In this study, the advanced statistical copula model was used on the kidney failure datasets obtained from Adama Hospital Medical College. This complex statistical model was used to investigate the correlation between the failure times. The comparisons of the models were done using the AIC and BIC. As a result, the log-logistic-Clayton copula model was found to be the most accurate statistical model for describing kidney failure datasets. The Clayton Archimedean copula family based on the graphical diagnosis was the fittest model for our datasets. The Weibull, Gompertz, and log-logistic baseline marginal distributions were all assumed, however, the log-logistic marginal distribution fit the kidney failure datasets the best.

 The failure times of the right and left kidneys were found to be dependent in this investigation. This could be because a pair of kidneys share the same biological gene. This supports the theory that the failure times of the paired human organs are connected since they are derived from the same person.^11-14,16,17^

 Age factor was found to be a major predictor of kidney failure in the study. According to the study, the likelihood of kidney failure was higher among elderly patients than others. This could be because as people get older, their kidneys tissues shrink, and their ability to function declines. This is consistent with an earlier research.^24-26^ Similarly, the sex of the patients was found to be a significant factor in their kidneys failure. According to the study, male patients were more likely to have kidney failure. This could be related to the men’s higher testosterone levels which impair the kidney function. This is also supported by the research undertaken in Japan^27^and Canada.^28^ One factor that significantly predicted the patient’s kidney failure was a family history of kidney disease. According to the previous research,^10,29-31^ patients with a family member diagnosed with a kidney illness, had a higher risk of kidney failure. The findings of this study demonstrated that individuals who were overweighed (obese) had a higher risk of kidney failure, which was consistent with the previous research.^32^ This could be due to the fact that the additional weight causes the kidney to work harder and filter wastes at a higher rate than usual. This additional work raises the risk of kidney disease over time. Diabetic individuals were shown in this study to be more likely to have kidney failure, which agreed with the previous research.^4,33,34^ This could indicate a problem with blood glucose (sugar). High blood sugar levels destroy the kidney’s millions of microscopic filtration units over time. As a result, kidney failure can occur. Furthermore, hypertension was found to be a determining prognostic factor for kidney failure in the patients in this investigation. Hypertensive patients were more likely to have kidney failure, according to the study. This could be related to the fact that uncontrolled high blood pressure can constrict, weaken, or stiffen the arteries near the kidneys over time. And then, these arteries will be unable to carry enough blood to the renal tissue due to their impairment. This result was also in accordance with the previous studies.^35-37^

 The advanced statistical copula model was used in this work, which was particularly intriguing when event times’ endpoints were dependent. Despite the strengths, there were also significant limitations to this study. Due to the use of the secondary data, socio-demographic characteristics such as marital status, occupation, religion (particularly), and the patients’ educational levels were not included in this study because they were not recorded.

## Conclusion

 The best statistical model for describing the kidney failure datasets was the log-logistic Clayton Archimedean copula model. The patients’ kidney failure risk factors included being a male, older adult, obese, hypertensive, diabetic, and also having a family history of kidney disease. The dependence between the patient’s time to the right and left kidneys failure was strong. Since hypertension, diabetes, and obesity were all risk factors for kidney failure, so lowering blood pressure, lowering sugar levels, and losing weight may help to prevent kidney failure. Because the failure of one kidney predicts the failure of the other, it is preferable to treat the failed one before it worsens.

**Highlights**

- The prevalence of at least one kidney failure was 39.4%.
- Hypertensive patients were more likely than non-hypertensive patients to have kidney failure.
- The times to (right and left) kidneys failure were highly correlated.

## Acknowledgment

 The authors gratefully acknowledge Adama Hospital Medical College for the provision of the data.

## Conflict of interest

 The authors declare that there was no conflict of interest in this study.

## Ethics approval and consent to participate

 Ethical approval was obtained from the Institutional Research Ethics Review Committee of the Jimma University College of Natural Sciences. A letter of support was written to Advanced Healthcare Management Corporation (AHMC). The authors submitted an official letter to AHMC. After clarifying the purposes of the study, the secondary data were obtained from all subjects and/or their legal guardian(s) for the participated cases who were children under 16. All methods were carried out following the relevant guidelines and regulations. Respondents had the right not to participate or withdraw from the study at any stage.

## Funding

 There was no direct fund for this study.
